# Evaluating an early social communication intervention for young children with Down syndrome (ASCEND): results from a feasibility randomised control trial

**DOI:** 10.1186/s40814-024-01551-y

**Published:** 2024-10-05

**Authors:** Vesna Stojanovik, Emma Pagnamenta, Sarah Sampson, Rachel Sutton, Benjamin Jones, Victoria Joffe, Kate Harvey, Elena Pizzo, Sarah Rae

**Affiliations:** 1 University of Reading, Reading, UK; 2 University of Exeter, Exeter, UK; 3 University of Essex, Colchester, UK; 4 University College London, London, UK; 5 Oxford Healthcare Foundation Trust, Oxford, UK

**Keywords:** Down syndrome, Intervention, Social communication, Language, Randomised controlled trial

## Abstract

**Background:**

This paper reports the results from a feasibility trial of an early parent-delivered social communication intervention for young children with Down syndrome (‘ASCEND’). The intervention focuses on developing children’s early social communication skills, in particular responding to shared attention. The aim was to inform the feasibility of running a full-scale trial through National Health Service (NHS) Speech and Language Therapy (SaLT) services, to assess whether the intervention is effective in improving language skills before children with Down syndrome start school.

**Methods:**

This was a two-arm feasibility randomised controlled trial (RCT), with 1:1 randomisation stratified by trial site, comparing the intervention plus standard NHS SaLT provision with standard NHS SaLT alone. We recruited 20 children with Down syndrome aged between 11 and 36 months through 3 NHS SaLT services, 19 of whom were randomised (10 — intervention group, 9 —control group). Pre- and post-intervention and 6-month follow-up assessments included language, social communication skills, adaptive behaviour, quality of life (parents and children), parental anxiety and depression. The intervention was parent delivered with parents having access to SaLT services and the research team during the intervention. Data were collected on recruitment and retention, standard care, treatment fidelity, acceptability of the intervention by the parents and speech and language therapists, feasibility of collecting health economic measures and suitability of the primary outcome measure.

**Results:**

The sample was sufficient for a feasibility study. The intervention (manual, support, materials) was positively received by the participating parents. Speech and language therapists also evaluated the acceptability of the intervention positively. Treatment fidelity which was measured by completion of weekly parent diaries and two adherence phone call was acceptable as 100% of the parent diaries were returned, over 90% of the parental diaries were completed correctly and 100% of adherence phone calls were completed. Retention was acceptable at 84% overall. The preliminary health economic data suggest that this intervention will be low cost. The sample size calculation suggests that 290 participants would need to be recruited, with 228 having a complete data set, for a full RCT.

**Conclusion:**

Based on recruitment, retention and treatment fidelity, as well as the acceptability of the intervention to parents and speech and language therapists, a full-scale trial would be feasible in order to assess the effectiveness of the intervention.

**Trial registration:**

ISRCTN13902755, registered on 25th August 2020, http://www.isrctn.com/ISRCTN13902755

**Supplementary Information:**

The online version contains supplementary material available at 10.1186/s40814-024-01551-y.

## Key messages

- What uncertainties existed regarding the feasibility?

We had uncertainties regarding study recruitment and retention of participants and whether parents would be willing for their child to be randomised to a control group, how acceptable the intervention would be for parents and for speech and language therapists, whether the intervention would be delivered as described in the protocol and how many participants we would need for a fully powered randomised controlled trial.

- What are the key feasibility findings?

The recruitment and retention rates are acceptable, recruitment was undertaken mainly through NHS SaLT services and through local charities, over 90% of parents were willing for their child to be randomized to the control group, the intervention package was positively evaluated by parents and speech and language therapists, and 290 participants with Down syndrome (with a view to having 228 complete the trial) are required, for a fully powered RCT.

- What are the implications of the feasibility findings for the design of the main study?

Based on our feasibility study outcomes, we conclude that a full-scale RCT is possible to assess the effectiveness of an early parent-delivered social communication intervention for young children with Down syndrome in optimising language development. The intervention is well suited for parents to deliver at home given the young age of the children, and this mode of delivery is in line with paediatric SaLT services.

## Background

Down syndrome is a genetic condition which results from an extra chromosome 21. Recent reports estimate a prevalence of 25.4 per 10,000 total births in England [1]. Down syndrome is the most common genetic cause of learning disability [2]. Most children with Down syndrome have difficulties acquiring speech and language, which often has adverse effects on communication skills. Evidence suggests that language ability at school entry can predict later psychosocial, educational and academic outcomes [3, 4], and that early language skills are primary indicator of child well-being [5]. It is therefore crucial that children with Down syndrome are provided with opportunities to advance their language and communication skills as early as possible. There is evidence from other clinical populations that shows early interventions can optimise language and communication outcomes [6, 7]. Given that Down syndrome can be diagnosed at birth, or even prenatally, interventions to optimise children’s language and communication outcomes can start very early.Children acquire language in the context of social interactions with others. Before children produce their first words, they acquire early social communication skills which are precursors for language [8–10]. Shared attention skills are fundamental early social communication skills acquired between 6 and 12 months of age. These skills allow the child and parent/caregiver to simultaneously focus on the same object or event, which in turn provides an opportunity for the parent/caregiver to name the object or event and for the child to learn a new word. The more a child responds to the parent’s/caregiver’s bids for shared attention, the more language input the child receives [11], and good quality and quantity of language input are essential for successful language acquisition [12–15]. Evidence suggests that how well a child responds to the parent/caregiver’s bids for shared attention is an important predictor of later language outcomes for children with Down syndrome [9, 16].

Reviews of interventions available which focus on the development of early social communication skills in young children with Down syndrome [17, 18] conclude that the current evidence base is of low quality due to low number of studies, heterogenous data and outcome measures and moderate to high risk of bias across studies. Our preliminary work [19, 20] shows that an early intervention focusing on social communication skills, and particularly on responding to shared attention, can lead to better language outcomes in young children with Down syndrome. The children, aged between 17 and 23 months with Down syndrome in the intervention group (*n* = 16), who had a 10-week intervention delivered jointly by a researcher and a parent, had significantly higher receptive vocabulary scores on the Reading Communicative Development Inventory (R-CDI) [21] 12 months after the intervention, compared to an age-matched control group of children with Down syndrome who did not receive this type of intervention.

The aim of the current feasibility study was to estimate the parameters to inform a future randomised controlled trial that will evaluate whether the early social communication intervention focusing on shared attention skills plus standard care is more effective than standard care alone for enhancing the language and early communication skills and family health outcomes for young children with Down syndrome aged 11 to 36 months.

The feasibility study’s *objectives* were as follows:

- Determine whether parents of children with Down syndrome are willing to be recruited as part of the study and be randomised.
- Determine the acceptability of the intervention to speech and language therapists (SaLTs).
- Determine the effectiveness of recruitment of children with Down syndrome by SaLTs.
- Identify different routes to identifying eligible children with Down syndrome (paediatricians, health visitors, SaLTs, charities).
- Estimate follow-up rate and adherence to intervention.
- Inform the measurement of health economic outcomes and resource implications of a parent-delivered intervention.
- Estimate the standard deviation of the primary outcome measure to inform a sample size calculation for a full trial.

## Method

### Feasibility trial design

The current study was a two-arm randomised controlled trial (RCT) which investigated the feasibility of carrying out a definitive RCT to evaluate the effectiveness of an early social communication intervention in addition to standard NHS speech and language therapy (SaLT) (compared with standard NHS SaLT alone) for young children with Down syndrome. The protocol was developed in line with the Standard Protocol Items: Recommendations for Interventional Trials [22] and published [23]. The results are reported in line with the CONSORT extension to pilot and feasibility trials [24].

### Setting

The study was conducted in three NHS sites in England, providing SaLT services across three geographical regions: Berkshire Health NHS Foundation Trust (BHFT), Oxford Health NHS Foundation Trust (OHFT) and North-East London NHS Foundation Trust (NELFT), and each site had a principal investigator. All assessments were conducted remotely using either online or paper questionnaires, with support by telephone. This was in response to the COVID-19 pandemic when most face-to-face services stopped. The protocol was amended so that there was no face-to-face contact between the research team and the participants and their families.

### Public and patient involvement (PPI)

The study protocol was developed with the help of our PPI representatives, which consisted of two parents of children with Down syndrome. They contributed to the finalising of the procedure, improving the readability of the parent manual and actively contributing to all decision-making regarding the feasibility trial as members of the Trial Steering Group which also included a highly specialist paediatric speech and language therapist, all three principle investigators, an independent statistician, an independent clinical research consultant, a developmental and heath psychologist with an interest in child development, a professor of developmental clinical psychology with a track record of clinical trials, the clinical trial manager and a representative of the sponsor (Berkshire Health Foundation Trust).

### Participants

**a) Children with Down syndrome and their parents/carers**

Children with Down syndrome and their families were recruited through SaLT services in BHFT, OHFT and NELFT who distributed information sheets and consent forms about the study to the parents/carers of every child with Down syndrome between the ages of 11 and 36 months on their caseloads. It was up to the parents/carers to decide whether one parent or both parents/carers participated and delivered the intervention. Parents who either declined to participate or did not engage with the research project were invited by their child’s SaLTs to give their reasons for not participating.

### Eligibility criteria

#### Inclusion criteria

1. Parent or guardian willing and able to provide informed consent on behalf of participant
2. Confirmed diagnosis of trisomy 21 (Down syndrome)
3. Male or female child, 11 to 36 months old at study entry
4. Parent/guardian has the literary and language skills needed to use the parent intervention manual.
5. The participant is not currently taking part or due to take part in a language-based intervention study.

#### Exclusion criteria

1. Children with comorbid conditions (for example autism spectrum disorder) as determined by the principal investigator for each NHS site
2. Any reason that may hinder participation, such as complex health issues requiring repeated hospital admissions
3. Prior knowledge of the intervention as specified in the parent manual

**b) Speech and language therapists**

SaLTs were recruited through clinical and professional networks and current NHS sites to take part in an interview on parent-delivered interventions, the acceptability of the intervention from a SaLT service delivery point of view and their views on clinical trials.

#### Inclusion criteria

1. Currently practising SaLT in the UK with a paediatric caseload (duration of practising with a paediatric caseload was not considered)
2. Currently working within the NHS or having recently worked within the NHS (not more than 2 years have passed since last NHS post)

### Procedure and intervention

The SaLTs supporting the children who took part in the intervention attended a 1-h training session delivered by the research team on the main goals of the intervention and the different stages so that they could support the parents if needed. The SaLTs had the opportunity to ask any questions and to feed into the intervention materials prior to the participant recruitment. The SaLTs acted as a point of contact for the families, to support the parents/carers with delivering the intervention when needed.

The intervention is focused on promoting and supporting the development of early social communication skills and, in particular, the child’s ability to respond to shared attention. During the sessions, the parent used the toys provided to encourage their child to engage in shared attention. A shared focus of attention can be achieved through seven levels, depending on the child’s developmental stage. During the sessions and over the course of the 10 weeks, parents/carers progressed through different levels of responding to joint attention from level 1 (the adult gently puts the child’s hand on object to signal that the specific object is the focus of attention) to level 7 (the adult placed a toy outside the child’s visual field, and the child then followed the adult’s gaze to establish shared attention with the object) (see Appendix 6 for a full description of the different levels based on Whalen and Schreibman [25]). Once shared attention was established (through any of the levels), the adult used this as an opportunity to provide rich language input to the child by the following: (a) labelling the object/labelling activities around the object (e.g. it is a bus; it drives around); (b) describing the toys/objects in terms of colour, shape, size and noise they make and how we can play with the toy; and (c) inviting the child to play/interact with the object. The approach is based on the social-pragmatic account of language acquisition, which assumes that shared attention and understanding the intentions of another person are the prerequisite skills for language development [26]. The parents recorded every session they had with their child on the diary form provided (see Appendix 1) and sent weekly to the trial manager.

### Recruitment of children with Down syndrome and their families

SaLTs from participating NHS trusts identified potential participants (children with Down syndrome) and their parents by reviewing their caseloads against the inclusion criteria. They then introduced the study to the parents of all potentially eligible children at a routine appointment or via email/telephone call and provided the parent/caregiver with a participant information sheet giving details of what study participation would involve. They invited them to contact the research team if interested in participating or if they had questions. Parents/carers took the time they needed to consider participation and were able to delay study entry by a few weeks to fit in with other family commitments. Where possible, reasons for nonparticipation were gathered by the child’s SaLT.

### Sample size

The target sample size was 25 children with Down syndrome based on literature recommendation of a minimum of 24 participants for feasibility studies [27–29] in order to estimate a standard deviation (SD) for the purposes of informing a subsequent sample size calculation.

### Randomisation and allocation

After written consent was given by the children’s parents/carers and baseline assessments completed, the children with Down syndrome were randomised by the clinical trial manager or other designated team member via Sortition® (a secure web-based clinical trial randomisation software developed by the University of Oxford) using block randomisation to receive standard care (Control) or standard care plus the intervention (Intervention) in a 1:1 ratio, stratified by site, to account for regional differences in standard care. Following randomisation, the parents/carers were contacted by the research team, who explained their child’s study allocation, what that meant and what would happen next.

### Blinding

Due to the nature of the intervention (parent delivered), it was impossible to blind the parents and the children’s SALTs to group allocation. The research assistant, who administered the pre- and post-intervention measures and who entered all the data, was blind to group allocation and also did not have access to the intervention materials until the end of the project.

### Intervention group

Once randomisation was complete, the intervention manual (paper based and printed using non-tear paper), blank diary forms (Appendix 1), a bag of age-appropriate toys and links to short video demonstrations of the intervention were sent to the parents/carers in the intervention group. The video materials showed each step of the intervention via short clips (2–3 min long). Each video clip was clearly labelled as to which level of shared attention was being shown. Each video showed a parent–child interaction, and there was a commentary (as a subtitle on the screen) explaining what the parent is doing and how the child is responding. The intervention was designed to be delivered by parents/carers over a period of 10 weeks. The intervention was delivered in the child’s home by one or both of their parent(s)/carer(s) over 10 weeks. Parents/carers were advised to deliver it for 1 h in total each week, over three to six individual sessions. The sessions could last between 10 and 20 min, and parents/carers chose how to allocate the time.

Support to deliver the intervention from the child’s SaLT was available at the request of parents by telephone/email. Parents also had access to the principal investigator of each site and to the chief investigator. The SaLTs and investigators recorded all contacts from parents including duration and content of each contact.

Families continued to access standard NHS SaLT for the duration of the project. All contacts related to standard care were recorded, including the duration, number of contact points and activity type (assessment, advice, intervention/review).

### Control (comparator) group: standard care

The control group received standard NHS SaLT care for this patient group. Standard care varied depending on each individual child’s needs and on the pathway specific to each NHS site, ranging from two contacts per year to monthly contacts. This typically included assessment, advice being given to parents on how to support their child’s general communication skills, review and intervention on feeding and use of baby sign Makaton. The SaLTs recorded all contacts with the family for the duration of the study.

At the end of the 6-month follow-up period, all families in the control group were provided with the intervention manual, accompanying materials and video links and had access to their child’s SaLT and/or members of the research team for support with delivering the intervention.

It is important to note that standard care did not include receiving a structured manual on how to support their children’s early social communication skills including shared attention nor videos showing how a child’s responding to shared attention could be supported. Standard care was more holistic covering different aspects of child development including communication, whereas our intervention specifically focused on building the children’s early social communication skills and language through shared attention skills, and it was very structured.

### Measures

Assessments were administered at baseline, immediately post intervention (10–14 weeks after baseline) and follow up (6 months later) and scored by a research assistant blind to group allocation.

- **a) Language and communication**

#### Primary outcome measure

*I) Reading Communicative Development Inventory (R-CDI)* [21] is a widely used parental checklist which assesses receptive and expressive language based on the MacArtur-Bates Communicative Development Inventories [30–32]. Parents are asked to tick the words their child understands, understands and says or understands and signs. We computed children’s understanding of words and expressive language. Expressive language was measured by adding together all spoken words and signs the child was reported to use. For the bilingual children, a total vocabulary was computed which included a sum of all the words (spoken, signed and understood) in both/all of their languages. The CDI was chosen as the primary outcome measure because the aim of the intervention is to increase children’s language by increasing their vocabulary and this measure directly assesses expressive and receptive vocabulary. Parental reports of language (such as the CDI) are widely used because parents have extensive experience with their children in a variety of naturalistic settings [33]. The MacArthur-Bates Communicative Development Inventories have been widely used in theoretical studies and in studies of importance for public health [31, 32, 33). This measure has a reasonable predictive and concurrent validity. For example, children’s scores on the CDI at ages 2 and 3 correlate significantly positively with standardised receptive language measures [33]. Importantly, the concurrent validity of the CDI has been established for children with Down syndrome [34]. The measures of children’s language obtained on the CDI and standardised measures of language correlated strongly between 0.70 and 0.82.

#### Secondary outcome measure

*Ii) Communication and Symbolic Behaviour Scale (CSBS)* [35]: This is a norm-referenced standardised tool available as an online or paper questionnaire, completed by parents/carers. The Infant–Toddler Checklist (which is part of the CSBS) has sensitivity of 78% and specificity of 84% [35]. It assesses communicative functions, gestural communicative means, vocal communicative means, verbal communicative means, reciprocity, social-affective signalling, and symbolic behaviour. This measure was chosen as a secondary outcome measure because the intervention focuses on increasing children’s early social communication behaviours and these can be assessed using this scale. The measure has reasonable reliability and validity [35].

- **b) Quality of life**
- The Infant Toddler Quality of Life (ITQOL-SF47) is completed by parents/carers and is a measure of infant quality of life. It is a reliable, valid and precise measure, and it was found to exceed item-level scaling criteria [36]
- The Adult Quality of Life Questionnaire [37] is completed by parents/carers and is a measure of parent/carer quality of life. It is a simple non-standardised instrument suitable for use with adult carers that measures quality of life in eight separate domains: support for caring, caring choice, caring stress, money matters, personal growth, sense of value, ability to care and carer satisfaction. The questionnaire was developed by initially putting together information from a range of sources including review of the literature on carers, scales used in previous carer research, an expert informed panel, and with the involvement of carers.
- The Hospital Anxiety and Depression Scale [38] is completed by parents/carers and is a self-assessment measure of symptoms of anxiety and depression. The scale is fully described in Zigmond and Snaith [38]. The internal consistency was established by item-subscale correlations, and significant associations of between 0.76 and 0.41 for the anxiety scale and between 0.60 and 0.30 for the depression scale were reported. Subsequent studies established further the psychometric properties with Cronbach’s alpha of 0.93 for the anxiety scale and 0.90 for the depression scale [39]. The validity of the scale was found to be satisfactory by Clark and Fallowfield (1988) [40].

In addition, all parents/carers of participating children completed a demographic questionnaire devised by the research team at baseline which asked questions about parental age, employment status and education and also contained the *Vineland Adaptive Behaviour Scale* [41], which is a standardised measure used to assess the children’s general cognitive and adaptive abilities and has acceptable validity and reliability and is the assessment of choice for educational, clinical and research purposed. For full details and a critical evaluation of its psychometric properties, see Pepperdine and McCrimmon [42].

All questionnaires were completed by parents/carers using online links, or using paper copies posted to the participants, with support from a member of the research team if required. The *Communication and Symbolic Behaviour Scale (CSBS)* was administered over the phone.

### Adherence to the intervention and contamination

Adherence to the intervention was monitored by asking the parents/carers to complete a weekly diary (see Appendix 1). The diary provided information on how many sessions the parent/carer carried out with their child, their duration, the number of different toys used at each session, the level at which they were working and additional comments. The principal investigators contacted the parents/carers by telephone in weeks 4 and 8 to check adherence, with a window of + / − 7 days. They asked a standard set of questions specifically designed to obtain information on how closely the parents were following the manual, how often they carried out the intervention sessions and their duration, the range of toys they used and what they usually did in order to engage their child (see Appendix 2 for a standard set of questions asked).

Contamination was assessed at the following: (1) study entry for those who were randomised to the intervention group and (2) before the final follow-up for those randomised to the control group. Parents/carers were sent a short questionnaire (Appendix 3) asking them whether they were familiar with the social communication intervention, whether they had seen the materials or whether they had seen the intervention being carried out. There was no evidence of contamination during the trial based on the data provided by the questionnaires. Given that the trial was ongoing during the height of the COVID-19 pandemic, the opportunities for parents/carers who live in different regions, to share the hard copies of the manual and toys provided, were minimal. Importantly, none of the participating families reported having seen the intervention manual and materials prior to being provided with their own set of the study materials.

### Parent/carer satisfaction with the intervention

Parent/carer satisfaction with the intervention was evaluated via a brief questionnaire comprising six questions (see Appendix 4), the first five of which were closed questions and the sixth, asking for comments on the intervention, was open-ended. Only parents/carers who completed the intervention were sent the questionnaire.

### Acceptability of the intervention to SaLTs

We invited all the SaLTs who had been involved with the current study to take part in in an interview with a member of the research team. We also opened the call to a wider group of SaLTs who had not been involved in the current study across the three NHS sites and SaLTs in other areas of the UK working in paediatric services for children with complex needs to take part in an interview as we wanted to have a broad range of views on this type of intervention and in SaLTs being involved in clinical trials, not only those who had been involved in the project. We distributed the information sheet through the Down Syndrome Research Forum, professional networks, through social media and SaLT managers and personal contacts. We aimed to recruit a diversity of SaLTs specifically in relation to gender, ethnicity and geographical area. In the last 3 months of the study, SaLTs were interviewed to explore their views on a parent-delivered intervention for young children with Down syndrome and their views and willingness to participate in future RCTs.

A topic guide was developed by the researchers addressing the study aims and used flexibly following the lead of the interviewees (Appendix 5). Data were collected via one-to-one interviews conducted via MS Teams. These lasted between 25 and 30 min each. Researchers reviewed and edited the interview transcripts auto produced by MS Teams.

### Analysis

Descriptive statistics (socio-demographic, language and cognitive abilities, and health status) were collated and summarised. Parent/carers’ satisfaction with the intervention was also summarised.

#### Quantitative analyses

All statistical analyses were prespecified in a statistical analysis plan (SAP) which was agreed and signed off by the trial statistician and chief investigator prior to commencement of any analyses. As this study only aimed to address feasibility objectives, no formal hypothesis testing was undertaken to make between-group comparisons, but rather summary statistics were calculated by allocated group and overall at each time point. For each outcome, point estimates of standard deviations (SDs) as well as associated 60%, 80% and 95% confidence intervals (CIs) are presented in line with Browne’s recommendation to use the limit of the one-sided 80% CI for an SD obtained from a pilot study to inform subsequent sample size calculation [43].

#### Qualitative analyses

The data from the parental responses (*n* = 11) regarding their reasons for not taking part in the study were summarised.

The data from the interviews with the SaLTs on their views of a parent-delivered social communication intervention were analysed using reflexive thematic analysis [44], an approach to data analysis that enables patterns of meanings across a dataset to be developed. Data were coded inductively using an essentialist approach to report the experiences, meanings and reality of participants [45]. The third author familiarised herself with the data and generated codes using NVivo (Version 20.6.1.1137). Codes were then discussed with the first author and refined. Where disagreements arose, agreement was reached by consensus. The third author then grouped the codes into themes. These themes were discussed and refined by the first author.

## Results

### Participant recruitment and flow

The SaLTs from the 3 participating sites approached 38 eligible participants. Of these, 18 declined to participate. Of those who declined, 11 parents provided reasons for declining which are the following: illness in family and not the right time, child was too ill to participate, family expecting a new baby so no time for intervention, other children in the family were home-schooled (due to lockdown) so parents felt unable to spend extra time with their child with Down syndrome, preference for an SaLT to work with their child directly and one of the parents/carers was not keen on taking part.

**Fig. 1:**
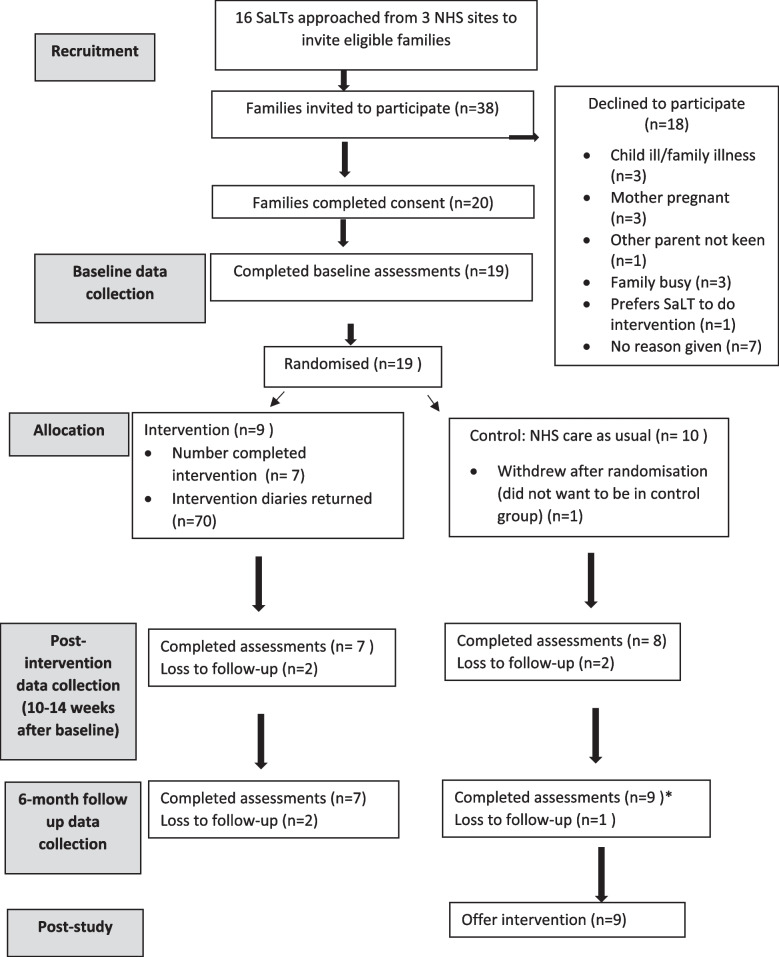
Study flow diagram. Note: NHS, National Health Service; SaLT, speech and language therapist. *One family was unable to complete Time 2 assessments, but the family was available to complete Time 3 assessments

Twenty parents completed the consent forms, and 19 families were randomised (1 family did not complete the baseline assessments and hence was not randomised). We did not recruit our target of 25 participants due to the restrictions arising from the COVID-19 pandemic which delayed recruitment and reduced the recruitment window by 6 months. The recruitment period varied between sites. Recruitment started between 9th September and 5th December 2020 and closed on 30th June 2021 with duration lasting between 7 and 10 months. Of the 19 participants, 9 were randomised to the intervention group and 10 to the control group. See Fig. 1 below for participant flow.

Although SaLTs were the obvious professionals to help recruit children with Down syndrome, not all young children with Down syndrome receive support from NHS SaLT services. We therefore explored other recruitment routes, including paediatricians, GPs, NHS networks of health professionals and charities which focus on supporting children with Down syndrome using clinical NHS networks and regional charities. Seventeen out of the 20 recruited families came through NHS SaLT services. Three families were recruited through charities and were advised to register with their local NHS SaLT service so that they could get support or alternatively would be supported by the principal investigator of the main NHS site (BHFT).

### Participants

The children had a mean age of 20.3 months, and 6 (32%) were female. Having completed the consent form and the baseline assessment, the parent of one child decided not to proceed with the study once they were informed that their child was randomised to the control group. However, given that baseline data were already collected and the participant randomised, their data are included. The sample’s baseline characteristics including age, gender, adaptive functioning and childcare are presented in Table 1. Characteristics of parents participating are presented in Table 2.

**Table 1:** Characteristics of the child participants in the intervention and control groups

n (%) unless otherwise stated	Intervention (*N* = 9)	Control (*N* = 10)	Overall (*N* = 19)
Mean (SD) [range] child’s age (months)	*n* = 9 20.6 (9.2) [8, 32]	*n* = 10 20.1 (9.2) [11, 36]	*n* = 19 20.3 (9.0) [8, 36]
n (%) child’s gender (female)	5 (56%)	1 (10%)	6 (32%)
n (%) > 3-week premature	3 (33%)	3 (30%)	6 (32%)
n (%) concerns about vision	1 (11%)	4 (40%)	5 (26%)
n (%) diagnosed mental, physical or emotional disability	8 (89%)	8 (80%)	16 (84%)
n (%) concerns about hearing	4 (44%)	8 (80%)	12 (63%)
n (%) history of ear infections	0 (0%)	0 (0%)	0 (0%)
n (%) hearing test	9 (100%)	10 (100%)	19 (100%)
Mean (SD) [range] hours in childcare per week	*n* = 9 12 (10.8) [0, 27]	*n* = 10 14.1 (16.8) [0, 50]	*n* = 19 13.1 (13.9) [0, 50]
**n (%) type of childcare**			
• *Family member*	2 (22%)	3 (30%)	5 (26%)
• *Child minder*	3 (33%)	0 (0%)	3 (16%)
• *Nursery*	2 (22%)	4 (40%)	6 (32%)
• *Nanny/Au pair*	0 (0%)	0 (0%)	0 (0%)
• *Other*	0 (0%)	0 (0%)	0 (0%)
• *N/A*	2 (22%)	3 (30%)	5 (26%)
n (%) receive portage	3 (33%)	6 (60%)	9 (47%)
n (%) receiving support from speech and language services	7 (78%)	10 (100%)	17 (89%)
**Mean standard score (SD) [range], Vineland Adaptive Behaviour Scale**			
• *Communication subdomain*	*n* = 9 39.8 (11.8) [24, 57]	*n* = 10 25.3 (11.3) [7, 39]	*n* = 19 32.2 (13.5) [7, 57]
• *Daily living skills subdomain*	*n* = 9 17.2 (8.7) [12, 38]	*n* = 10 9.0 (2.8) [5, 14]	*n* = 19 12.9 (7.4) [5, 38]
• *Socialisation subdomain*	*n* = 9 42.7 (6.8) [32, 55]	*n* = 10 33.1 (10.0) [13, 43]	*n* = 19 37.6 (9.7) [13, 55]
• *Overall score*	*n* = 9 99.7 (21.7) [68, 137]	*n* = 10 67.4 (21.4) [29, 92]	*n* = 19 82.7 (26.7) [29, 137]

**Table 2:** Parents’ characteristics

n (%) unless otherwise stated	Intervention (*N* = 9)	Control (*N* = 10)	Overall (*N* = 19)
Mean (SD) parent age (years)	*n* = 9 38.4 (3.6) [33, 44]	*n* = 10 37.3 (4.8) [30, 45]	*n* = 19 37.8 (4.2) [30, 45]
n (%) parent gender (female)	9 (100%)	10 (100%)	19 (100%)
**n (%) parent occupation status**			
• *Employed full time*	1 (11%)	4 (40%)	5 (26%)
• *Employed part-time*	5 (56%)	2 (20%)	7 (37%)
• *Self-employed*	1 (11%)	0 (0%)	1 (5%)
• *Unemployed*	1 (11%)	3 (30%)	4 (21%)
• *Employed (on parental leave)*	1 (11%)	1 (10%)	2 (11%)
**n (%) highest level of parent education**			
• *None*	0 (0%)	0 (0%)	0 (0%)
• *GCSEs/O-levels*	0 (0%)	0 (0%)	0 (0%)
• *A-levels*	2 (22%)	2 (20%)	4 (21%)
• *NVQ/HND*	0 (0%)	3 (30%)	3 (16%)
• *Degree*	5 (56%)	2 (20%)	7 (37%)
• *Postgraduate degree*	1 (11%)	2 (20%)	3 (16%)
• *Other*	1 (11%)	1 (10%)	2 (11%)
n (%) born in the UK	4 (44%)	7 (70%)	11 (58%)
Mean (SD) [range] if not born in the UK, number of years in the UK	*n* = 5 16.3 (5.5) [8.5, 23]	*n* = 3 21.0 (7.9) [15, 30]	*n* = 8 18.1 (6.4) [8.5, 30]
***Partner (other parent) characteristics***			
Mean (SD) [range] partner age	*n* = 9 37.8 (3.8) [33, 43]	*n* = 10 37.5 (6.4) [29, 47]	*n* = 19 37.6 (5.2) [29, 47]
n (%) partner gender (female)	0 (0%)	0 (0%)	0 (0%)
**n (%) partner occupation status**			
• *Employed full time*	6 (67%)	8 (80%)	14 (74%)
• *Employed part-time*	1 (11%)	1 (10%)	2 (11%)
• *Self-employed*	0 (0%)	1 (10%)	1 (5%)
• *Unemployed*	1 (11%)	0 (0%)	1 (5%)
• *Employed (on parental leave)*	0 (0%)	0 (0%)	0 (0%)
**n (%) highest level of partner education**			
• *None*	0 (0%)	1 (10%)	1 (5.3%)
• *GCSEs/O-levels*	0 (0%)	2 (20%)	2 (11%)
• *A-levels*	0 (0%)	0 (0%)	0 (0%)
• *NVQ/HND*	0 (0%)	2 (20%)	2 (11%)
• *Degree*	4 (44%)	3 (30%)	7 (37%)
• *Postgraduate degree*	4 (44%)	0 (0%)	4 (21%)
• *Other*	0 (0%)	0 (0%)	0 (0%)

Note: *GCSE* General certificate of secondary education, *NVQ* National vocational qualification

The results of the proposed outcome measures are presented in Table 3 below. Data are presented at baseline, immediately post-intervention (3 months after the baseline) and follow-up (6 months following the end of the intervention).

**Table 3:** Summary statistics for proposed outcome measures

*Mean (SD) [range] outcome*	*Baseline*			*Post-intervention*			*Follow-up*		
	*Intervention (N* = *9)*	*Control (N* = *10)*	*Overall (N* = *19)*	*Intervention (N* = *7)*	*Control (N* = *8)*	*Overall (N* = *15)*	*Intervention (N* = *7)*	*Control (N* = *9)*	*Overall (N* = *16)*
**RCDI**									
*Receptive language (RCDI-U)*	*n* = 9 127.3 (178.0) [1, 567]	*n* = 10 69.5 (59.7) [0, 181]	*n* = 19 96.9 (129.4) [0, 567]	*n* = 7 217.6 (146.5) [52, 372]	*n* = 8 55.6 (38.4) [9, 128]	*n* = 15 131.2 (130.1) [9, 372]	*n* = 7 229.7 (151.1) [71, 468]	*n* = 9 91.0 (62.8) [0, 173]	*n* = 16 151.7 (127.6) [0, 468]
*Expressive language (RCDI-E)*	*n* = 9 53.7 (84.0) [0, 268]	*n* = 10 11.1 (11.2) [0, 32]	*n* = 19 31.3 (60.7) [0, 268]	*n* = 7 51.4 (60.2) [0, 148]	*n* = 8 12.5 (14.2) [0, 34]	*n* = 15 30.7 (45.4) [0, 148]	*n* = 7 99.7 (75.1) [10, 217]	*n* = 9 26.4 (27.4) [0, 86]	*n* = 16 58.5 (63.8) [0, 217]
*RCDI total*	*n* = 9 131.2 (125.1) [4, 384]	*n* = 10 74.6 (57.1) [0, 156]	*n* = 19 101.4 (97.1) [0, 384]	*n* = 7 269.0 (167.1) [65, 474]	*n* = 8 68.1 (48.0) [9, 162]	*n* = 15 161.9 (154.5) [9, 474]	*n* = 7 323.3 (199.6) [65, 618]	*n* = 9 117.4 (78.9) [30, 259]	*n* = 16 207.5 (174.3) [30, 618]
**CSBS**				l					
*Social composite*	*n* = 9 18.0 (3.4) [11, 22]	*n* = 10 11.9 (2.8) [8, 17]	*n* = 19 14.8 (4.4) [8, 22]	*n* = 7 21.4 (1.6) [19, 23]	*n* = 8 13.9 (3.4) [10, 18]	*n* = 15 17.4 (4.7) [10, 23]	*n* = 7 21.1 (1.6) [19, 24]	*n* = 9 14.3 (3.9) [9, 22]	*n* = 16 17.3 (4.6) [9, 24]
*Speech composite*	*n* = 9 7.4 (2.1) [4, 10]	*n* = 10 4.7 (2.8) [0, 9]	*n* = 19 6.0 (2.8) [0, 10]	*n* = 7 8.6 (2.6) [5, 12]	*n* = 8 7.0 (1.8) [5, 9]	*n* = 15 7.7 (2.3) [5, 12]	*n* = 7 10.4 (2.2) [7, 13]	*n* = 9 6.7 (3.4) [1, 11]	*n* = 16 8.3 (3.4) [1, 13]
*Symbolic composite*	*n* = 9 10.8 (4.0) [4, 16]	*n* = 10 7.7 (3.2) [2, 13]	*n* = 19 9.2 (3.8) [2, 16]	*n* = 7 13.6 (2.9) [10, 17]	*n* = 8 9.8 (3.0) [6, 14]	*n* = 15 11.5 (3.5) [6, 17]	*n* = 7 14.1 (2.8) [9, 17]	*n* = 9 11.4 (3.6) [5, 16]	*n* = 16 12.6 (3.5) [5, 17]
*Total*	*n* = 9 36.2 (8.0) [21, 48]	*n* = 10 24.3 (7.4) [12, 33]	*n* = 19 29.9 (9.7) [12, 48]	*n* = 7 43.6 (5.5) [36, 49]	*n* = 8 30.6 (7.0) [21, 38]	*n* = 15 36.7 (9.1) [21, 49]	*n* = 7 45.7 (5.1) [38, 53]	*n* = 9 32.4 (7.5) [19, 42]	*n* = 16 38.3 (9.3) [19, 53]

Note: *RCDI* Reading Communicative Development Inventory — raw scores, *CSBS* Communication and Symbolic Behaviour Scale — raw scores

### Outcomes

The aim of this feasibility trial was to obtain descriptive statistics that can be used to calculate the sample size needed for a subsequent definitive trial. A summary of the descriptive statistics for the primary (RCDI) and secondary (CSBS) outcome measures is presented in Table 3 below.

### Acceptability of the intervention to salts

The final sample included 12 paediatric SaLTs (2 males, 10 females) with a range of experience and from a range of different NHS trusts in England. Six SaLTs had been part of the intervention study, and 6 had not. They all either currently worked for the NHS or had done so in the past 2 years. The interview data indicate that 11 out of the 12 SaLTs were supportive of a parent-delivered intervention focusing on early social communication skills for young children with Down syndrome. All SaLTs taking part agreed that parent-delivered interventions should be offered by SaLT services, and that such interventions were in alignment with existing provisions:

So that’s the way our service is now developing. It’s more focused on. You need to do these things at home and then contact us if you’ve still got concerns (SaLT X01)

So I work in early years in the NHS so all our interventions are, primarily are, parent led. (SaLT Y03)

Some SaLTs felt that alternative/additional interventions were necessary in addition to this intervention:

I don’t think that parent led intervention, working on play, etc., is enough for children with Down Syndrome […..] so I think that the evidence and my own experience shows that direct therapy, regular direct therapy for children with Down Syndrome does work… (SaLTZ05)

All SaLTs either have or would continue to recommend parent-delivered interventions to parents and were most likely to offer them to parents of children who were under 5. When asked about how the interventions they offer may be similar or different from our intervention, some similarities were identified (for example use of parent diaries, instructions given). The main difference identified was that our proposed intervention was much more structured.

It's not something that we've used as in such a structured way (SaLT Y03)

### Acceptability of the intervention to parents

This was assessed using a questionnaire which was sent to the parents in the intervention group only, once they had completed the study. A total of 90% parents reported that they were very satisfied with the intervention, one parent was fairly satisfied and one parent was neutral. All parents reported that they thought their child’s responding to shared attention had improved following the intervention. Most parents reported that their child’s language and communication had improved as a result of improvement in shared attention.

We found that he is trying to communicate more and getting our attention until he was able to get what he was asking for or wanting to do (Parent 7)

Definitely. For example she can keep eye contact better and can express her needs on a more understandable way (Parent 3)

Most parents reported seeing improvement in other areas of development in their child such as visual awareness, visual tracking, eye contact, copying of actions and gross motor skills.

Gross motor skills improved a lot: nearly running and jumping (Parent 1)

…Visual tracking and eye contact (Parent 5)

Most parents also reported that they had changed the way they communicate with their child after the intervention by using more descriptive language, providing more language input, encouraging their child to use different toys and using techniques learnt from the intervention in everyday situations.

…Trying to input more verbal language than Makaton signs (Parent 1)

I am giving more description and talking to him more since we started the intervention (Parent 4).

Parents were also asked for any other general comments and feedback. There was a mixture of comments with some helpful suggestions of what we may need to adjust in future, which included more instructions on what to do after the child had passed the final level and also the suitability of the intervention for children who were at the older end of our age range.

It was very beneficial intervention for both of us, thank you for giving us this valuable opportunity. We had a great support from the study team. They helped/guided us immediately when needed. (Parent 4)

The exercises were generally well explained in the manual, and the videos were helpful. Could potentially have more instructions about what to do if you complete the final level, unless it's ok to just practise whichever aspects of the previous levels you/your child wants to (which is what we did). (Parent 2)

It was a good and fun programme to see the way in which X understands and responds. (Parent 6)

I think X was too young at the beginning and found the repeats boring but it was not a problem after a few weeks. The programme should have been longer a bit because we needed to repeat certain weeks and could not reach level 10. We really enjoyed the sessions and the quality time we spent with each other while doing it. (Parent 3)

### Adherence to the intervention and contamination

Adherence phone calls (week 4 and week 8) were completed for seven of the nine families in the intervention arm. These seven families completed all follow-up assessments as well. There was acceptable evidence from the data collected in the telephone contacts that the parents were following the instructions in the manual and completing the different stages of the intervention as suggested by the manual. Seven out of nine families who were randomised to the intervention arm completed intervention diaries (78%), and all parents whose children completed all three assessments completed all diaries (100%) and all adherence calls. The mean length of session was 17 min (range 5 to 30 min), and parents spent 60 min per week on average with a range of 58 min to 64 min. Parents carried out on average 4 sessions per week, with a range of two to six sessions per week. This is in line with the instructions in the manual. Inspection of the parent-completed diaries showed that over 90% of the parents completed the diaries correctly. We did not collect diaries from the participants in the control arm.

### Estimate of the sample size of a future trial to evaluate the effectiveness of the early social communication intervention

The proposed primary outcome in a subsequent definitive trial is the total number of words (expressive, receptive and signed, across multiple languages if the participant is multilingual) as measured using the R-CDI [21], assessed at the primary endpoint of 6 months following the end of the intervention. In order to achieve 90% power to detect a between-group difference of 100 words at the 5% two-sided significance level, a total of 228 participants (114 per arm) will need to be followed up and provide valid outcome data at the primary endpoint. This assumes a standard deviation of the outcome of 231 words, which conservatively represents the upper limit of the 80% confidence interval of the standard deviation estimated from the feasibility study. In consultation with our PPI group and the currently available literature on how children with Down syndrome acquire language [46, 47] as well as the relatively simple nature of the intervention, it was agreed that 100 words would be a clinically important difference. An increase of 100 words in a child’s repertoire will bring a child with Down syndrome closer to the point of starting to combine words into sentences (the average 24-month-old child has 297 words and is combining words into sentences) [48]. A change of 100 words, on average, represents a minimum clinically important change in the primary outcome and characterises a meaningful improvement in language development compared to typical development in this population. Assuming a loss to follow-up of 20% (similar to the feasibility study), the final indicative recruitment target in a definitive trial is therefore a total of 290 participants. Assuming an average of 20 children per site over a recruitment period of 24 months, we would need 15 sites.

Several scenarios were considered (see Table 4 below).

**Table 4:** Sample size scenarios

Standard deviation	Target difference	Standardised effect size	Required participants followed up	Target sample size (assuming 20% loss to follow-up)
***Base case***				
231	100	0.43	228	286
***Vary SD***				
129	100	0.78	72	90
143		0.70	88	110
154		0.65	102	128
174		0.57	130	164
210		0.48	188	236
270		0.37	310	388
***Vary target difference***				
231	80	0.35	354	444
	90	0.39	280	350
	110	0.48	188	236
	120	0.52	158	198

Note: *SD* Standard deviation

### Health economics/health outcomes

Three measures were employed to look at parent/carer and infant quality of life. These measures were chosen and agreed jointly by parents of children with Down syndrome during a focus-group interview, and these parents did not take part in the intervention (as they had older children with Down syndrome). The following measures were used: Adult Carer Quality-of-Life Questionnaire (ACQOL) [37], the Infant and Toddler Quality-of-Life Questionnaire (ITQOL-SF47) [36], and the Hospital Anxiety and Depression Scale (HADS) [38]. Table 5 below shows the summary statistics of the health outcomes measures.

**Table 5:** Summary statistics of health outcome measures

*Mean (SD) [range] outcome*	*Baseline*			*Post-intervention*			*Follow-up*		
	*Intervention (N* = *9)*	*Control (N* = *10)*	*Overall (N* = *19)*	*Intervention (N* = *7)*	*Control (N* = *8)*	*Overall (N* = *15)*	*Intervention (N* = *7)*	*Control (N* = *9)*	*Overall (N* = *16)*
**Adult Carer Quality of Life**									
Total quality sum score (raw score)	*n* = 9 79.9 (18.9) [49, 105]	*n* = 10 79.3 (19) [43, 108]	*n* = 19 79.6 (17.9) [43, 108]	*n* = 7 87.7 (16.1) [39, 109]	*n* = 8 74.3 (23.3) [40, 112]	*n* = 15 77.5 (20.7) [39, 112]	*n* = 7 79.3 (19.7) [51, 108]	*n* = 9 73.4 (18) [42, 96]	*n* = 16 76 (18.2) [42, 108]
**Infant–Toddler Quality of Life** (standard score)	*n* = 9 63.3 (23.8) [30, 100]	*n* = 10 67 (18) [30, 85]	*n* = 19 65.3 (20.4) [30, 100]	*n* = 7 72.1 (23.6) [30, 100]	*n* = 8 62.2 (16.2) [30, 85]	*n* = 15 66.6 (19.7) [30, 100]	*n* = 6 67.5 (22.1) [30, 85]	*n* = 9 67.8 (18.9) [30, 85]	*n* = 15 67.7 (19.4) [30, 85]
**HADS** Depression overall score	*n* = 9 3.7 (3.2) [0, 9]	*n* = 10 6.8 (4.5) [1, 12]	*n* = 19 5.3 (4.2) [0, 12]	*n* = 7 3.3 (2.4) [0, 6]	*n* = 9 7.4 (4.9) [2, 18]	*n* = 16 5.6 (4.4) [0, 18]	*n* = 7 5.8 (5) [0, 15]	*n* = 9 7.2 (2) [5, 11]	*n* = 16 6.3 (4) [0, 15]
**HADS** Anxiety overall score	*n* = 9 6.3 (3.9) [3, 14]	*n* = 10 6.8 (4.5) [1, 12]	*n* = 19 8.5 (5.5) [1, 14]	*n* = 7 4.3 (2) [1, 7]	*n* = 9 9.8 (6.4) [4, 20]	*n* = 16 7.4 (5.6) [1, 20]	*n* = 7 7.7 (5.7) [2, 18]	*n* = 9 12 (4) [7, 17]	*n* = 16 9.3 (5.4) [2, 18]

Note: *HADS* Hospital Anxiety and Depression Scale

During the feasibility study, we estimated that the intervention cost is around £142–£174 per child. This includes 1 h per child of SaLT time (the cost can vary between £82 and £114 per hour, PSSRU 2021 [49], and the material used is estimated to cost approximately £60 per child (this includes a canvas bag, colour-printed manual on special non-tear paper, seven different toys and postage and packing)). We estimate that to run a full trial with 228 children, the cost of the intervention would be between £32,376 and £39,672. This does not include the cost for the families. The cost to families will be calculated in a full trial.

### Adverse events and safety

No adverse events were reported.

## Discussion

The aim of this study was to explore the feasibility of running a full-scale RCT to evaluate the effectiveness and cost-effectiveness of an early parent-delivered social communication intervention for young children with Down syndrome in addition to standard SaLT care compared to standard SaLT care only. To this end, in this feasibility trial, we investigated if we could recruit enough children with Down syndrome and parents/carers, the most relevant recruitment pathways and the retention rate, how acceptable a parent-delivered intervention would be for parents/carers and also for SaLTs, adherence to protocol, ways to assess cost effectiveness and participant numbers for a full-scale RCT.

### Recruitment and retention

We originally planned to recruit 25 children with Down syndrome over a period of 12 months. However, due to the COVID-19 pandemic and the interruption and delays this caused to the trial, our three sites were only open for recruitment for between 7 and 10 months (rather than the planned 12 months), and we recruited 20 participants. We are reasonably confident that if we had had the 3 sites recruiting for the full 12 months, we would have reached our target of 25 participants. Our sample size calculation suggests that we would need to recruit 290 participants with a view to having 228 participants with a full data set for a full RCT. This means 15 sites are needed which will recruit on average 20 participants each over a period of 24 months, and this we believe is feasible. We also established during the feasibility study that the most effective recruitment route was via SaLT services and charities supporting families with children with Down syndrome.

Retention was monitored by the clinical trial manager who followed up families and who was responsible for letting the research assistant know when families were due to be sent assessments. Of the original 19 families who were randomised and who completed baseline assessments (time point 0), 15 families completed the second assessment (time point 1), which is retention of 79%, and 16 families competed the third assessment (time point 2), which means retention was 84% overall. Retention of 70% is the minimum specified if a study is going to be included in a Cochrane review [50].

### Acceptability to parents and SaLTs

Both parents of children with Down syndrome and SaLTs were generally very positive about the acceptability of the intervention. For the SaLT services, barriers identified included mainly time and resource. The positive perspective of the intervention by parents is likely a reflection of the positive active engagement of our PPI group, which resulted in the information given to parents being clear, provided in ‘parent-friendly’ format, accessible and easy to follow. The high acceptability of the intervention for SaLTs was likely facilitated by to the ongoing conversations and engagement with the NHS sites about the intervention delivery and how well it would fit within current models of delivery of early interventions prior to the study commencing. For the parents, barriers identified were mainly the child’s age as some parents felt that their children were either too young or too old to fully benefit from the programme. Also, some parents felt that we could have suggested more activities for children who managed to go through all levels of the intervention before the end of the 10 weeks.

### Treatment fidelity and adherence

Based on the adherence phone calls in weeks 4 and 8, and submitted parent diaries, we are satisfied that the intervention was delivered by the parents with high fidelity and as described in the manual and videos. This is based on the fact that 100% of the parent diaries (for the parents who provided complete data sets for all time points) were returned, over 90% of the diaries were completed correctly and the adherence phone calls were all completed and did not identify any issues. It is very important that the materials are self-explanatory, and that parents can use them autonomously, with minimal support from SaLT services. All parents who responded to the post-intervention questionnaire stated that the materials were clear and easy to follow.

The SaLTs who participated in the interviews also all commented that the materials provided were clear and straightforward. Although this did not come up during the study, we are aware that our current materials need to be updated so that they are inclusive and representative of the population as a whole in terms of diversity, and we will be updating these before we run a full-scale trial.

### Health economic measures and cost-effectiveness

The economic analysis results show that the intervention can be delivered at a reasonable cost. We recommend that a detailed cost data collection is conducted in a full trial to make sure all NHS resources are included. We also recommend collecting data on the private costs for the families (e.g. private cost for other health services, adaptations, special toys, time off work, transport costs). In this study, we have identified three health outcome measures of effectiveness, and we recommend that these can be translated in utility values to be used in a future cost-utility analysis.

### Limitations

A potential limitation arises from the self-report nature of the adherence measurement. Although this was deemed to be feasible, in the absence of direct observation of the families, we are limited in our ability to fully evaluate whether or not parents were implementing the intervention strategies accurately. Another possible limitation is the fact that only mothers/female carers completed the questionnaires; however, we did not collect data on who administered the intervention sessions at home. We will collect this information in a subsequent trial as there are established differences between how fathers/male carers and mothers/female carers communicate to young children, and it is important to record this information.

## Conclusion

This feasibility study is the first-step in the development of an evidence-based theoretically driven early social communication intervention for young children with Down syndrome, bridging a gap in the current evidence base for intervention for very young children. Based on the outcomes, and specifically given the rates of recruitment, retention and data completeness, and based on the finding that the study procedures were acceptable for parents and SaLTs, a full-scale trial appears feasible and warranted.

### Trial status

Ethical approval was granted on 4th August 2020 for the amended protocol (South Central — Berkshire Research Ethics Committee ref.: 19/SC/0572, IRAS Project ID: 252332). Recruitment opened on 9th September 2020 for BHFT, 10th November for NELFT, and 9th December for OHFT. Recruitment closed on 30th June 2021 for all sites.

## Supplementary information

Additional file 1: Appendix 1: Weekly diary.

Additional file 2: Appendix 2: Adherence to intervention.

Additional file 3: Appendix 3: Contamination questionnaire.

Additional file 4: Appendix 4: Parent satisfaction questionnaire.

Additional file 5: Appendix 5: Topic guide for interviews with SaLTs about intervention acceptability

Additional file 6: Appendix 6: Intervention levels (1-7) based on Whalen & Schreibman (2003).

## Data Availability

The dataset resulting from the study is currently being stored on a secure server at the University of Reading. https://researchdata.reading.ac.uk/470/.
