# Supplementary material for: Evaluating an early social communication intervention for young children with Down syndrome (ASCEND): results from a feasibility randomised control trial
Source: Pilot Feasibility Stud. 2024 Oct 5;10:127. doi: 10.1186/s40814-024-01551-y (PMC11453083; doi:10.1186/s40814-024-01551-y)
Supplement: Supplementary file 1 — Additional file 1: Appendix 1: Weekly diary. [file 40814_2024_1551_MOESM1_ESM.docx]

Appendix 1: Weekly diary

HOW TO COMPLETE THE WEEKLY DIARY

Dear parents,

Here are some pointers on how to complete your weekly intervention diary.

1. As you can see, we have allowed for up to 6 sessions per week as some of you may choose to work with you child 3 times a week, 20 mins each time, or 4 times per week, 15 mins each time, or 6 times per week, 10mins each time. You could also do a combination of these. For example, your first session could be 20 mins, your next 4 sessions could be 10 minutes each. It’s ok as long as the total time adds up to 1 hour.
2. It is also possible that your child is ready to move from one level to the next within the week. That’s ok, please indicate in the relevant box which level you are working at.
3. Please also indicate if your child needed to prompt them to respond (out of 5 attempts)
4. When the child is able to respond to 4 out of 5 attempts unprompted, put a tick next to that toy and use different toys (as the child needs to be able to respond unprompted on 4 out of 5 attempts to 5 different toys).
5. Please remember that your child is ready to move to the next level when they are able **to respond without prompts** on 4 out of 5 attempts with 5 different toys. If your child responded on 4 out of 5 attempts with prompts, they need more practice.
6. Also please write which toys you have used.
7. We have also included a ‘Comments Box’ for each week, so that you can put in anything that you have observed. For example: a child may have got really obsessed with one specific toy, or your child spontaneously said/signed a new word during the session, or you struggled to understand what you were supposed to do.

See below for an example completed diary for ONE week and parent’s comments.

| **Child’s name:** Jane | | | | **Week of intervention (1-10):** 1 | | |
| --- | --- | --- | --- | --- | --- | --- |
|  | **Session 1**  **Date (dd/mm/yyyy)**  20/01/2020 | **Session 2**  **Date (dd/mm/yyyy)**  22/01/2020 | **Session 3**  **Date (dd/mm/yyyy)**  23/01/2020 | **Session 4**  **Date (dd/mm/yyyy)**  26/01/2020 | **Session 5**  **Date (dd/mm/yyyy)** | **Session 6**  **Date (dd/mm/yyyy)** |
| **Level (1-7)** | 1 | 1 | 1 | 1 |  |  |
| **Session length (min)** | 15 | 10 | 20 | 15 |  |  |
| **Number of toys used (up to 5)** | 2 | 1 | 3 | 2 |  |  |
| **Toy 1** | Car | Car ✓ | Ball ✓ | Bus ✓ |  |  |
| Child responded unprompted | 0 | 4 | 4 | 4 |  |  |
| Child responded when prompted | 1 | 1 | 1 | 1 |  |  |
| Child did not respond when prompted | 2 | 0 | 0 | 0 |  |  |
| **Toy 2** | Ball |  | Bus | Train |  |  |
| Child responded unprompted | 0 |  | 3 | 3 |  |  |
| Child responded when prompted | 1 |  | 2 | 2 |  |  |
| Child did not respond when prompted | 1 |  | 0 | 0 |  |  |
| **Toy 3** |  |  | Train |  |  |  |
| Child responded unprompted |  |  | 2 |  |  |  |
| Child responded when prompted |  |  | 3 |  |  |  |
| Child did not respond when prompted |  |  | 0 |  |  |  |
| **Toy 4** |  |  |  |  |  |  |
| Child responded unprompted |  |  |  |  |  |  |
| Child responded when prompted |  |  |  |  |  |  |
| Child did not respond when prompted |  |  |  |  |  |  |
| **Toy 5** |  |  |  |  |  |  |
| Child responded unprompted |  |  |  |  |  |  |
| Child responded when prompted |  |  |  |  |  |  |
| Child did not respond when prompted |  |  |  |  |  |  |
| **Summary of week:**  Move to next level: YES NO ✓ | | | | | | |
| Any other comments: | | | | | | |

As you can see, in the first session the parent used 2 different toys (car, ball), in the second session 1 toy (ball) as it was a short session, in session 3 the parent used 3 different toys (ball, bus, train) and in the last session, the parent used 2 different toys (bus, train).

In session 1, the parent used prompts 3 times with the first toy (car) to get a response from the child and 2 prompts for the second toy (ball), however the child only responded to one of these.

In session 2, the parent used prompts once to get a response from the child, whereas on 4 out of 5 attempts, the child responded without prompts. Hence the parent has put a tick next to the word ‘car’

In session 3, the parent used one prompt for the first toy (ball) to get a response from the child and the child responded unprompted to the other 4 attempts. Hence the parent has put a tick next to the word ‘ball’. For the second toy (bus) the parent used a prompt on 2 out of 5 occasions and for the third toy (train), the parent used a prompt on 3 out of 5 occasions.

In session 4, the parent used one prompt for the first toy (bus) to get a response from the child and the child responded unprompted to the other 4 attempts. Hence the parent has put a tick next to the word ‘bus’. For the second toy (train), the parent used a prompt on 2 out of 5 occasions.

The child is not able to move from level 1 to level 2 yet because the child was able to respond unprompted on 4 out of 5 requests for 3 different toys. So in week 2, the parent will continue to work on level 1.
