# Supplementary material for: Evaluating an early social communication intervention for young children with Down syndrome (ASCEND): results from a feasibility randomised control trial
Source: Pilot Feasibility Stud. 2024 Oct 5;10:127. doi: 10.1186/s40814-024-01551-y (PMC11453083; doi:10.1186/s40814-024-01551-y)
Supplement: Supplementary file 2 — Additional file 2: Appendix 2: Adherence to intervention. [file 40814_2024_1551_MOESM2_ESM.docx]

Appendix 2: Adherence to intervention

**Adherence and compliance with manual- telephone call**

| **Date of call** |  |
| --- | --- |
| **Participant ID** |  |
| **Name of person making the call** |  |
| **Intervention week** | - Week 4 - Week 8 |
| **Q1: How many sessions have you done with your child during the past week?** | - 1 - 2 - 3 - 4 - 5 - 6 - 7 - More than 7 |
| **Q2: How long was each session approximately?** | - 1-5 minutes - 6-10 minutes - 11-15 minutes - 16- 20 minutes - More than 20 minutes |
| **Q3: Which level have you focussed on during the last week?** | - 1 - 2 - 3 - 4 - 5 - 6 - 7 |
| **Q4: Tell me about the last session - what did you do and which toys did you use** |  |
| **Q5: How did your last session go?** | - My child engaged with the toys and we completed all the steps - My child engaged with the toys and we completed most of the steps - My child engaged with the toys and we completed some of the steps - My child engaged briefly with the toys - My child did not engage with the toys |
| **Q6: How has your child responded to the sessions during the past week?** | - Very engaged - Mostly engaged - Sometimes engaged - Rarely engaged - Not engaged |
| **Q7: What do you usually do if your child doesn’t engage?** |  |
| **Q8: Have you completed your weekly diary for the past week?** | - Yes - No |
| **Q9: Have you got any other comments?** | - Yes - No   If yes, then elaborate here |
