# Supplementary material for: Evaluating an early social communication intervention for young children with Down syndrome (ASCEND): results from a feasibility randomised control trial
Source: Pilot Feasibility Stud. 2024 Oct 5;10:127. doi: 10.1186/s40814-024-01551-y (PMC11453083; doi:10.1186/s40814-024-01551-y)
Supplement: Supplementary file 3 — Additional file 3: Appendix 3: Contamination questionnaire. [file 40814_2024_1551_MOESM3_ESM.docx]

Appendix 3: Contamination questionnaire

CON01_Assessment of contamination

| Date (DD/MM/YYYY): | Participant ID: |
| --- | --- |
|  |  |

Have you heard about the social communication intervention? Yes / No

If so how?

…………………………………………………………………………………………………

Have you seen the intervention manual? Yes / No

Have you seen the intervention being done? Yes / No
