# Supplementary material for: Evaluating an early social communication intervention for young children with Down syndrome (ASCEND): results from a feasibility randomised control trial
Source: Pilot Feasibility Stud. 2024 Oct 5;10:127. doi: 10.1186/s40814-024-01551-y (PMC11453083; doi:10.1186/s40814-024-01551-y)
Supplement: Supplementary file 4 — Additional file 4: Appendix 4: Parent satisfaction questionnaire. [file 40814_2024_1551_MOESM4_ESM.docx]

Appendix 4: Parent satisfaction questionnaire

Evaluating an early social communication intervention for young children with Down Syndrome (ASCEND): a feasibility study.

**Parent questionnaire – satisfaction of intervention**

1. How satisfied are you generally with the intervention programme? (please circle)

Very satisfied Fairly satisfied Neutral Fairly dissatisfied Very dissatisfied

1. Do you think your child’s responding to shared attention has improved since starting the intervention?

Yes No

Comments:__________________________________________________________

1. Do you think any improvement in shared attention has influenced your child’s speech/language/communication skills?

Yes No

Comments:__________________________________________________________

1. Have you noticed any improvement in other areas of your child’s development?

Yes No

Comments: _________________________________________________________

1. Have you changed anything about your communication with your child since the start of the intervention?

Yes No

Comments:__________________________________________________________

1. Any general feedback would be greatly appreciated – what worked well, what could be improved, why are you satisfied/dissatisfied with the programme?
