# Supplementary material for: Evaluating an early social communication intervention for young children with Down syndrome (ASCEND): results from a feasibility randomised control trial
Source: Pilot Feasibility Stud. 2024 Oct 5;10:127. doi: 10.1186/s40814-024-01551-y (PMC11453083; doi:10.1186/s40814-024-01551-y)
Supplement: Supplementary file 5 — Additional file 5: Appendix 5: Topic guide for interviews with SaLTs about intervention acceptability [file 40814_2024_1551_MOESM5_ESM.docx]

Appendix 5: Topic guide for interviews with SaLTs about intervention acceptability

TOPIC GUIDE FOR INTERVIEWS WITH SPEECH AND LANGUAGE THERAPISTS

One of the objectives of the Feasibility study is to assess the acceptability of the intervention to speech and language therapists and effectiveness of recruitment of children with DS by speech and language therapists. To achieve this objective, all Speech and Language Therapists (SaLT) who had facilitated recruitment and/or the delivery of the intervention during the feasibility study, as well as other SaLTs from Oxfordshire, Berkshire and other counties with paediatric caseloads are invited to participate in an interview with a member of the research team. From this pool of participants, we will purposively sample so that all SaLTs who had supported the delivery of our intervention during the feasibility study (10-12) take part, and SaLTs who were not involved in the feasibility study with a range of specialisms are also represented.

The interview will be conducted either over the phone or face to face. It will be audio-recorded for later transcription and analysis.

The following topic guide will be used during the interviews.

1. The Research Assistant introduces themselves and thanks the participant for agreeing to take part. The interview will last about 30 mins
2. Ask if the SaLT took part in the Feasibility Trial

YES NO

**If they answered YES, ask the following questions:**

- What did you think of the Parent manual provided (in terms of usability and quality of information)?
  - Do you think that parents *could* use the information provided in the manual? (probe why could/couldn’t use the information)
  - Do you think parents used the information in the manual? (probe why would/wouldn’t use the information)
  - What do you think worked well?
  - What did not work well?
- What did you think about the training the research team provided?
- Was it useful, and if so how?
  - Was it necessary, and if so why and how? (probe if there were bits that were more or less necessary)
  - What do you think worked well?
  - What do you think didn’t work well?
  - Would you have been able to understand the information in the Parent Manual without the training provided?
- Do you think that this type of parent-led intervention should be offered and supported by SaLT services? Probe:
  - Can you tell me more about why you think that
  - Are there other types of intervention that SaLT services could offer that would be better?
  - Are other services (not SaLT) better placed to offer this type of intervention?
  - What do you think parents think about SaLT services offering this kind of intervention?
- What do you think about Clinical Trials?
  - Do you think they are a good way of evaluating interventions? (probe why/why not)
  - Do you think they are practical? (probe why/why not)
  - Do you think they are a good thing for patients and/or practitioners? (probe why/why not)
- Would you be interested in being part of a clinical trial which is looking at implementing a parent-led intervention for young children with Down Syndrome?
  - Would you find the prospect of being involved in a clinical trial appealing (probe why/why not)
  - Is there anything that would make you reluctant to be involved in a clinical trial?

**If the SaLT answered NO for question 2, then ask the following:**

- What do “parent-led interventions” mean to you? Here, we mean an intervention which is entirely delivered by a parent following a manual, with occasional support by an SaLT), even it is confirming SaLTs understanding (*aim is to ensure a shared understanding of parent led interventions*)
- What do you think about this kind of parent-led intervention?
- Have you ever recommended to a parent a parent-led intervention in your paediatric practice? Probe:
  - What made you recommend it?
  - Was it a *parent led intervention* like the one in this study? (probe how was it similar/different)
  - Do you think it was a success?
  - Would you recommend a parent-led intervention in future? (probe why/why not, if yes to whom/in what circumstances)
- What do you think about Clinical Trials?
  - Do you think they are a good way of evaluating interventions? (probe why/why not)
  - Do you think they are practical? (probe why/why not)
  - Do you think they are a good thing for patients and/or practitioners? (probe why/why not)
- Would you be interested in being part of a clinical trial which is looking at implementing a parent-led intervention for young children with Down Syndrome?
  - Would you find the prospect of being involved in a clinical trial appealing (probe why/why not)
  - Is there anything that would make you reluctant to be involved in a clinical trial?
