# Supplementary material for: Evaluating an early social communication intervention for young children with Down syndrome (ASCEND): results from a feasibility randomised control trial
Source: Pilot Feasibility Stud. 2024 Oct 5;10:127. doi: 10.1186/s40814-024-01551-y (PMC11453083; doi:10.1186/s40814-024-01551-y)
Supplement: Supplementary file 6 — Additional file 6: Appendix 6: Intervention levels (1-7) based on Whalen & Schreibman (2003). [file 40814_2024_1551_MOESM6_ESM.docx]

Appendix 6: Intervention levels (1-7) based on Whalen & Schreibman (2003)

| Level 1 | Response to hand on object (adult gently puts child’s hand on object to draw child’s attention to it) |
| --- | --- |
| Level 2 | Response to tapping object (adult taps object to draw child’s attention to it) |
| Level 3 | Response to object activation (adult uses a mechanical toy which moves/makes a noise to draw child’s attention to it) |
| Level 4 | Eye contact – the adult encourages child to make eye-contact while also engaging with a toy/object |
| Level 5 | Following points – choice of two toys - the aim is for the child to follow adult’s eye-gaze by following adult’s pointing |
| Level 6 | Following points in a book - The goal is for the child to follow the adult’s eye-gaze (line of regard) by following adult’s points in a book |
| Level 7 | Following points around the room - The goal is for the child to follow the adult’s eye gaze (line of regard) by following the adult’s point to something that is outside of their visual field e.g. behind them or something they have to look up or down for, or to the left and right of the child. |
